# Déterminants de la survie des enfants âgés de 6 mois à 15 ans, infectés par le VIH et suivis dans la ville d’Ebolowa au Cameroun de 2008 à 2018

**DOI:** 10.11604/pamj.2020.37.308.25829

**Published:** 2020-12-03

**Authors:** Ginette Claude Mireille Kalla, Valery-Gustave Mve Mve, Nelly Kamgaing Noubi, Marcelle Nina Ehouzou Mandeng, Marie Claire Okomo Assoumou, Francois Xavier Mbopi-Keou, Francisca Monebenimp

**Affiliations:** 1 Faculté de Médecine et des Sciences Biomédicales, Université de Yaoundé I, Yaoundé, Cameroun,; 2 Service de Pédiatrie, Centre Hospitalier et Universitaire de Yaoundé, Yaoundé, Cameroun

**Keywords:** VIH, enfant, survie, Ebolowa, Cameroun, HIV, child, survival, Ebolowa, Cameroon

## Abstract

**Introduction:**

la survie des enfants infectés par le VIH demeure un défi dans les pays en voie de développement. Au Cameroun, la mortalité liée au VIH chez les enfants de moins de 15 ans en 2018 était de 20%. Paradoxalement, la région du Sud Cameroun, malgré une séroprévalence élevée chez les enfants de 4,1% et une couverture en traitement antirétroviral faible de l´ordre de 64%, ne fait pas partie des régions du Cameroun les plus touchées par la mortalité pédiatrique liée au VIH/SIDA. L´objectif de ce travail était de déterminer le taux de survie et identifier ses déterminants chez les enfants âgés de 6 mois à 15 ans, infectés par le VIH.

**Méthodes:**

une étude de cohorte à collecte de données rétrospective et prospective a été menée de janvier 2008 à décembre 2018 dans trois formations sanitaires prenant en charge les enfants VIH positifs, à Ebolowa dans la région du Sud Cameroun. L´étude s´est faite en deux temps, une phase de collecte rétrospective pour la sélection des dossiers médicaux des enfants VIH positifs répondant aux critères d´inclusion dans les registres de consultation, et une phase de collecte prospective qui nous a permis d´avoir auprès des parents, les informations sur le devenir des enfants. Un consentement éclairé parental a été obtenu au cours de cette deuxième phase. Les données sociodémographiques, cliniques, paracliniques, thérapeutiques, ainsi que le devenir des enfants ont été collectées. Les temps moyens de survie, ainsi que les facteurs associés à la survie ont été déterminés à l´aide du modèle de Kaplan Meier. La régression à risque proportionnel de Cox, nous a permis d´identifier les déterminants de la survie. Notre critère de jugement était le décès. Le niveau de significativité a été fixé à 5%.

**Résultats:**

au total, 186 enfants ont été enrôlés. La durée médiane de suivi était de 18,5 mois. Le taux de survie était de 66,7%. La majorité des décès (67%) est survenue avant le sixième mois de suivi. Après analyse multivariée, l´âge inférieur à 2 ans [aHR: 18,6 (6,48-53,59); p=0,001], l´anémie sévère [aHR: 7,69 (1,02-57,9); p=0,04], et la présence d´infections opportunistes [aHR: 4,52 (2,51-8,14); p=0,05] étaient indépendamment et significativement associés à la survie.

**Conclusion:**

en plus du traitement antirétroviral précoce, un bon suivi clinique et paraclinique est nécessaire pour améliorer la survie des enfants infectés par le VIH.

## Abstract (en)

**Introduction:**

survival of HIV-infected children is a challenge in developing countries. In Cameroon, HIV-related mortality among children under the age of 15 in 2018 was 20%. Paradoxically, the Southern Cameroon region, despite high seroprevalence among children (4.1%) and low antiretroviral therapy coverage (around 64%), is not among the regions of Cameroon most affected by HIV/AIDS-related pediatric mortality. The purpose of this study was to calculate survival rate and to identify its determinants in HIV-infected children aged 6 months-15 years.

**Methods:**

we conducted a retrospective, prospective cohort study data-collection in three health care facilities specialized in treating HIV-positive children in Ebolowa, South Cameroon from January 2008 to December 2018. The study was conducted in two phases, a retrospective collection phase for the selection of medical records of HIV-positive children that met inclusion criteria in consultation registries and a prospective collection phase in which we collected information from parents about the future of children. Informed parental consent was obtained during this second phase. Socio-demographic, clinical, paramedical, therapeutic data as well as data about the future of children were collected. Mean survival time and factors associated with survival were determined using the Kaplan Meier model. Cox proportional hazards regression allowed for the identification of survival determinants. Evaluation criterion was the death. Significance level was set at 5%.

**Results:**

a total of 186 patients were enrolled in the study: the average follow-up period was 18.5 months. Survival rate was 66.7%. The majority of deaths (67%) occurred before the sixth month of follow-up. After multivariate analysis, an age less than 2 years [aHR: 18.6 (6.48-53.59); p=0.001), severe anemia [aHR: 7.69 (1.02-57.9); p=0.04) and the presence of opportunistic infections [aHR: 4.52 (2.51-8.14); p=0.05] were independently and significantly associated with survival.

**Conclusion:**

in addition to early antiretroviral therapy, good clinical and paraclinical monitoring is needed to improve the survival of HIV-infected children.

## Introduction

La survie des enfants infectés par le virus de l´immunodéficience humaine (VIH) demeure un problème de santé publique à l´échelle mondiale, notamment dans les pays en voie de développement et surtout en Afrique subsaharienne [1]. En 2008, 91% de nouvelles infections à VIH chez les enfants ont été répertoriées dans cette région [2]. En 2016, il a été noté 91% de décès liés au VIH/SIDA chez les adolescents [3].

En 2017, selon le Comité National de Lutte contre le Sida (CNLS), la prévalence générale du VIH/SIDA était estimée à 3,1% [4]. L´ONUSIDA estime d´ailleurs, que le Cameroun reste l´un des pays d´Afrique subsaharienne les plus touchés et que les enfants de moins 15 ans représenteraient 7,96% de personnes vivant avec le VIH. En 2018, 15,000 à 21,000 décès étaient liés au VIH parmi lesquels, 2400 à 4600 enfants de moins de 15 ans soit 20% de décès [1].

Des stratégies de riposte y ont été mises en place pour réduire cette mortalité. Ces mesures étaient axées sur la prévention, particulièrement la prévention de la transmission mère enfant (PTME) initiée en 1996 [5]. Le traitement antirétroviral (TAR) a été gratuit depuis 2007 [6]. Le diagnostic précoce par *polymerase chain reaction (PCR)* a été effectif depuis 2008 [5]. En outre, le TAR immédiat a débuté en 2011 [7]. Grâce à ces efforts, l´on a observé un recul de l´épidémie chez les enfants de 2010 à 2018 avec une diminution de nouvelles infections et une baisse de la mortalité respectivement de 44% et 45% [1].

Cette amélioration globale, est cependant inégalement répartie dans les différentes régions du pays. En effet, la région du Sud-Cameroun présente depuis plusieurs années les séroprévalences au VIH les plus élevées. Estimée dans la population générale à 5,2% en 2017 [4], chez les enfants nés de mères séropositives au VIH, la séroprévalence était d´environ 4,1% avec une couverture en TAR d´environ 64% [8], bien loin de l´atteinte de l´objectif 90-90-90 prévus à l´horizon 2020 [9].

De nombreuses études faites reconnaissent la place fondamentale qu´occupe la thérapie antirétrovirale pour la survie des enfants infectés par le VIH. Une méta-analyse réalisée dans plusieurs pays africains en 2011 montrait le bénéfice du traitement antirétroviral sur la survie des enfants infectés par le VIH comparativement à celle des enfants ne recevant pas de traitement [10]. Nyunt *et al*. en Asie avaient trouvé qu´entre 2006 et 2016, la survie globale des enfants sous TAR était de 86% [11]. Malgré le faible taux de couverture en ARV, la région du Sud Cameroun ne figure pas parmi les régions pour lesquelles on enregistre le plus de décès pédiatriques liés au VIH/SIDA au Cameroun [5,7]. Fort de ce constat, nous nous sommes posé la question de savoir ce qui, en dehors du TAR, expliquerait la survie des enfants infectés par le VIH dans la ville d´Ebolowa? D´où notre objectif qui était de déterminer le taux de survie et identifier ses déterminants chez ces enfants.

## Méthodes

Une étude de cohorte à collecte rétrospective (dans les dossiers médicaux) et prospective (suite à une rencontre avec les parents) a été menée dans trois formations sanitaires à file active dans la ville d´Ebolowa, chef-lieu de la région du Sud Cameroun sur une période allant de Janvier 2008 à Décembre 2018. La population d´étude était constituée d´enfants âgés de 6 mois à 15 ans révolus, infectés par le VIH et suivis dans ladite ville. Dans chaque formation sanitaire, les registres ont été consultés et les dossiers médicaux des enfants répondant à nos critères d´inclusion ont été retenus. Dans ces dossiers, les données sur les variables sociodémographiques, cliniques, paracliniques et thérapeutiques ont été recueillies. A partir du contact téléphonique des parents ou tuteurs retrouvé dans les dossiers, ces derniers ont été contactés afin d´obtenir leur consentement éclairé et avoir des informations sur le devenir des enfants.

**Procédures:** après approbation du Comité d´Ethique et de la Recherche de la Faculté de Médecine et des Sciences Biomédicales de l´Université de Yaoundé I, une autorisation de recherche auprès du délégué régional de la santé publique du sud a été obtenue. Les registres des enfants suivis pour infection à VIH ont été consultés. Tous les enfants dont l´âge était compris entre 6 mois et 15 ans révolus dont le diagnostic avait été posé entre le 1^er^ janvier 2008 et le 31 décembre 2018 ont été retenus. Les variables sociodémographiques, cliniques, paracliniques, thérapeutiques, ainsi que les informations sur le devenir des enfants obtenues auprès des parents après consentement parental, ont été collectées sur des fiches techniques conçues à cet effet. Les registres de laboratoire, ainsi que les registres de ravitaillement en ARV ont également été consultés. Était considéré comme perdu de vue, tout enfant non retrouvé après recherche active dans la communauté.

**Analyse des données:** les variables sociodémographiques, cliniques, paracliniques, thérapeutiques, ainsi que le devenir des enfants VIH positifs et suivis à Ebolowa ont été étudiées. Les aspects descriptifs ont été présentés sous forme de tableaux ou figures avec, les effectifs, pourcentages, médianes, moyennes et écart-types. Le modèle de Kaplan Meier a permis de déterminer les temps moyens de survie des enfants infectés par le VIH. Les courbes de Kaplan Meier nous ont permis d´identifier les facteurs associés à la survie. Les variables dignes d´intérêt avec p<0,1 à l´analyse bivariée ont été introduites dans le modèle de régression à risque proportionnel de Cox. Les déterminants indépendants de la survie ont été identifiés en utilisant les rapports de risques ajustés à un intervalle de confiance de 95% et une valeur p<0,05. Le critère de jugement était le décès. L´analyse statistique des données a été effectuée en utilisant les logiciels Microsoft Excel et SPSS version 17.

**Considérations éthiques:** un consentement éclairé des parents a été obtenu pour la phase de collecte de données prospective. L´anonymat a été conservé.

## Résultats

**Caractéristiques sociodémographiques des enfants parents et tuteurs:** du 1^er^ janvier 2008 au 31 décembre 2018, 186 enfants ont été retenus dont 124 (66,7%) vivants et sous TAR, 45 (24,2%) décédés et 17 (9,1%) perdus de vue. Quatre-vingt-douze (49,5%) étaient de sexe masculin et 94 (50,5%) de sexe féminin soit un sexe ratio de 0,98. La médiane d´âge était de 84,5 mois avec des extrêmes allant de 7 à 191 mois. La tranche d´âge la plus représentée était celle de 5-10 ans (38,2%). La majorité des enfants 117 (63%) vivaient en zone urbaine. Soixante et seize d´entre eux (40,9%) étaient orphelins d´au moins un parent. La majorité 132 (71%) était scolarisée (Table 1).

**Table 1:** caractéristiques sociodémographiques des enfants infectés par le VIH et suivis à Ebolowa

Variables	Effectifs (n)	Pourcentages (%)
**Tranches d’âge (années)**		
≤2	22	11,8
(2-5)	45	24,2
(5-10)	71	38,2
(10-16)	48	25,8
**Sexe**		
Masculin	92	49,5
Féminin	94	50,5
**Résidence**		
Urbain	118	63
Rural	69	37
**Statut d’orphelin**		
Oui	76	40,9
Non	110	59,1
**Scolarisation**		
Oui	132	71
Non	21	10,3
Pas encore	33	17,7

La majorité des mères 130 (69,9%) étaient vivantes et avaient un âge compris entre 16 et 61 ans. La tranche d´âge des mères la plus représentée était celle de 17-35 ans (67%). La sérologie VIH des mères était positive pour 112 d´entre elles (86,1%) et 93 (83%) étaient sous TAR. Seulement 12 mères avaient un travail rémunéré (9,2%), 62 d´entre elles vivaient en couple (47,7%), 51 mères (39,2%) étaient célibataires et 17 étaient veuves (13,1%). La majorité des pères étaient vivants (n=84, 45%), avec un âge variant entre 22 et 71 ans. La tranche d´âge la plus représentée celles de 35-55 ans (75%). La sérologie VIH était connue positive pour 23 (27,3%) d´entre eux et seulement 15 (71,4%) étaient sous TAR. Vingt-et-un avaient un travail rémunéré (25%), 60 soit 61,4% vivaient en couple, 13 (15,5%) étaient célibataires et 11 (13,1%) étaient veufs.

**Caractéristiques cliniques, paracliniques et thérapeutiques:** le diagnostic de l´infection à VIH a été fait par sérologie pour la majorité 155 (83,3%) et seulement 31 (16,7%) ont été diagnostiqués précocement par PCR. Pour 143 (76,9%) d´entre eux, le diagnostic s´est fait au stade de Sida maladie. Plus de la moitié était déjà à un stade clinique avancé de la maladie au moment du diagnostic avec respectivement 81 (43,5%) au stade III et 19 (10,2%) au stade IV. La charge virale initiale avait été faite chez 35 (18,8%) enfants. Le dosage du taux de CD4 au début de la prise en charge n´a été disponible que pour 89 enfants sur les 186 soit 47,8% et 50 (56,2%) d´entre eux étaient en immunodépression sévère. Cent-deux enfants ont bénéficié d´une NFS initiale et la majorité était anémiée 88 (86,3%), dont 28 (27,5%) étaient en anémie sévère. Tous les enfants ont été mis sous ARV, 174 (93,5%) étaient sous prophylaxie à base de cotrimoxazole. Le ravitaillement en ARV était de 100%, 50-99% et <50% respectivement pour 117 (62,9%), 52 (30%) et 17 (9,1%) enfants (Table 2).

**Table 2:** caractéristiques cliniques, paraclinique et thérapeutique à l'initiation du traitement antirétroviral

Variables	Effectifs (n)	Pourcentages (%)
**Méthodes de diagnostic**		
PCR	31	16,7
Sérologie	155	83,3
**Classification clinique OMS**		
Stade I	42	22,6
Stade II	44	23,6
Stade III	81	43,5
Stade IV	19	10,2
**Comorbidités liées au VIH**		
Oui	64	34,4
Non	122	65,6
**Stade immunologique**		
Pas d'ID	20	22,5
ID modérée	7	7,9
ID avancée	12	13,5
ID sévère	50	56,2
**Anémie**		
Pas d'anémie	14	13,7
Légère	29	28,4
Modérée	31	30,4
Sévère	28	27,5
**Ravitaillement en ARV (%)**		
100	117	62,9
50-99	52	30
0-49	17	9,1

ID: immunodépression; ARV: antirétroviraux

**Suivi:** la durée du suivi était comprise entre 1-157 mois avec une médiane de 18,5 mois. Un changement de protocole est survenu chez 12 (6,5%) enfants. Les raisons de ce changement étaient l´échec clinique pour 5 (41,7%) enfants et la rupture de stock des ARV pour 4 (33,3%) enfants. Seuls 32 (17,2%) ont présenté au moins une infection opportuniste au cours du suivi. Les contrôles de charge virale et de dosage de CD4 au cours du suivi n'ont été faits que chez 8 (4,3%) et 18 (9,8%) enfants respectivement.

**Figure 1:**
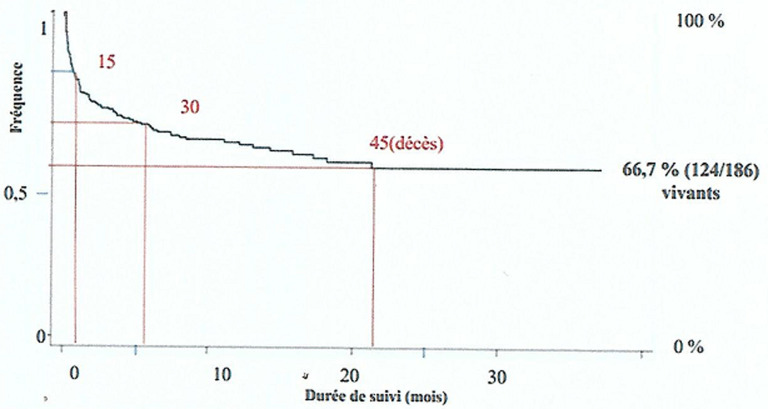
courbe globale de survie des enfants infectés par le VIH et suivis à Ebolowa

**Devenir:** sur les 186 enfants retenus, 124 étaient en vie au moment de l´étude soit un taux brut de survie de 66,7% (Table 3). Des 45 (24,2%) enfants décédés, deux tiers l´ont été avant le 6^e^ mois de suivi et la courbe globale de survie a montré une probabilité de survie meilleure après le 22^e^ mois de suivi (Figure 1).

**Table 3:** devenir des enfants infectés par le VIH et suivis à Ebolowa

Variables	Effectifs (n)	Pourcentages (%)
**Devenir**		
Vivants	124	66,7
Décédés	45	24,2
Perdus de vue	17	9,1
Total	186	100

**Facteurs associés à la survie:** l´âge était associé significativement à la survie (p<0,0001) avec des temps moyens de survie de 8,5 ± 1,8 mois, 32,4 ± 3,2 mois, 66,3 ± 6,9 mois et 105,8 ± 16,1 mois pour les enfants de <2 ans, 2-5 ans, 5-10 ans et >10 ans respectivement. La scolarisation était associée significativement à la survie (p=0,004) avec un temps moyen de survie de 79,6 ± 5,4 mois pour les enfants scolarisés et de 60,7 ± 14,7 mois pour ceux non scolarisés. La présence de comorbidités liées au VIH était associée significativement à la survie (p=0,005). Le temps moyen de survie des enfants qui ne présentaient pas de comorbidités était de 79,0 ± 5,3 mois et de 57,4 ± 12,9 mois pour ceux qui en présentaient.

Une association significative a été retrouvée entre le stade clinique au moment du diagnostic et la survie (p<0,0001). Les temps moyens de survie étaient de 84,1 ± 9,7 mois, 51,0 ± 4,5 mois, 78,9 ± 14,5 mois et 26,2 ± 8,1 pour les stades cliniques I, II, III et IV respectivement. L´anémie était un facteur associé à la survie (p=0,003). Les temps moyens de survie étaient de 41,4 ± 9,5 mois pour les enfants avec anémie sévère, 95,6 ± 16,6 mois pour ceux avec anémie modérée et 75,5 ± 9,3 mois pour les enfants avec anémie légère.

**Figure 2:**
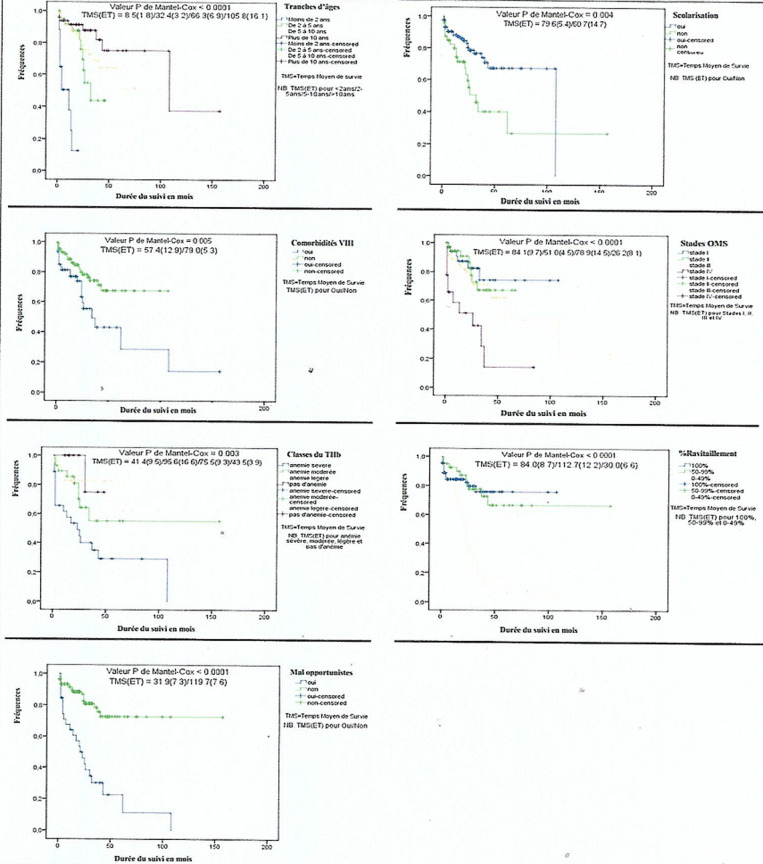
facteurs associés à la survie des enfants infectés par le VIH et suivis à Ebolowa

Le ravitaillement en ARV était associé significativement à la survie (p<0,0001) avec un temps moyen de survie de 84,0 ± 8,7 mois pour les enfants qui avaient un pourcentage de ravitaillement de 100%, 112,7 ± 12,2 mois pour des ravitaillements compris entre 50-99% et 30,0 ± 6,6 mois pour ceux dont le ravitaillement était < à 50%. Une association significative a été retrouvée entre les maladies opportunistes et la survie (p<0,0001) avec un temps moyen de survie de 119,7 ± 7,6 mois pour les enfants qui n´en avaient pas et 31,9 ± 7,3 mois pour ceux qui en avaient (Figure 2).

**Figure 3:**
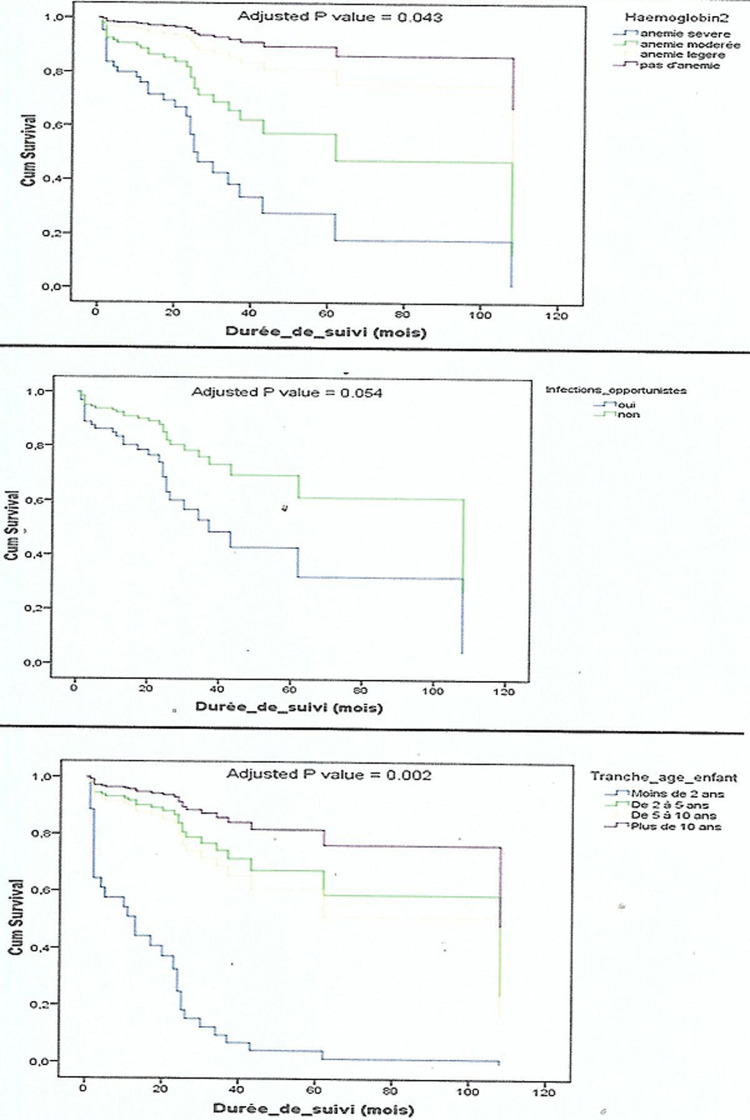
courbes de survie ajustées des enfants selon les tranches d’âge, classes d’hémoglobine et maladies opportunistes

**Analyse multi variée:** l´âge <2 ans [HR: 15,62 (3,1-79,7), p=0,001], l´anémie sévère [HR: 11,5 (1,1-125,3) p=0,04], la présence de maladies opportunistes [HR: 2,33 (0,98-5,5), p=0,05] ont été retrouvés comme facteurs indépendants significativement associés à la survie (Table 4 and Figure 3).

**Table 4:** déterminants de la survie des enfants infectés par le VIH et suivis à Ebolowa

Variables	Effets non ajustés HR (IC â 95%)	p value	Effets ajustés HR (IC â 95%)	p value
**Tranches d'âge (années)**		**<0,0001**		
≤2	18,6 (6,48-53,59)	<0,0001	15,62 (3,1-79,7)	0,001
(2-5)	2,9 (1,08-7,78)	0,034	1,96 (0,37-10,5)	0,432
(5-10)	1,92 (0,79-4,66)	0,152	2,48 (0,74-8,33)	0,143
(10-16)	1	1	1	1
Scolarisation (Oui/Non)	0,43 (0,24-0,78)	0,006	0,96 (0,33-2,8)	0,94
Méthodes diagnostiques (PCR/Sérologie)	1,78 (0,91-3,47)	0,09	0,3 (0,07-1,13)	0,08
Comorbidités liées au VIH (Oui/Non)	2,26 (1,23-4,1)	0,007	0,73 (0,2-2,64)	0,63
**Stade clinique OMS**		**<0,001**	**0,53**	
Stade IV	6 (0,7-35,2)	0,001	4,57 (0,12-33,76)	0,062
Stade III	2,23 (1,09-16,58)	0,002	1,67 (0,13-13,5)	0,16
Stade II	3,3 (0,94-11,64)	0,002	0,88 (0,17-17,29)	0,09
Stade I	1	1	1	1
Comorbidités non liées au VIH (Oui/Non)	0,56 (0,29-1,07)	0,08	0,68 (0,17-2,6)	0,587
**Anémie**		**0,008**		**0,043**
Sévère	7,69 (1,02-57,9)	0,048	11,5 (1,1-125,3)	0,04
Modérée	3,15 (0,4-24,94)	0,277	5,04 (0,47-53,3)	0,18
Légère	1,96 (0,23-16,9)	0,539	1,89 (0,19-19,1)	0,59
Pas d'anémie	1			
Ravitaillement en ARV (%)		<0,0001	0,19	
0-49	11 (3,12-29,3)	<0,0001	1,95 (0,79-17,5)	0,07
50-99	5,21 (0,19-37,48)	<0,0001	0,3 (0,54-7,64)	0,08
100	1	1	1	1
Maladies opportunistes (Oui/Non)	4,52 (2,51-8,14)	<0,0001	2,33 (0,98-5,5)	0,05

## Discussion

Au total, sur la période allant de 2008 à 2018 nous avons répertorié dans les registres de consultation, 186 enfants infectés par le VIH et suivis à Ebolowa. Le taux brut de survie au moment de l´étude était de 66,7%. Ce taux est inférieur au taux global de survie de 86% retrouvé par Nyunt *et al*. en 2018 au Myanmar. Cette différence pourrait s´expliquer par le fait que la majorité (83%) des enfants de leur étude résidait en zone urbaine, avaient plus de 200 CD4/mm^3^ avant la mise sous TAR et étaient sous prophylaxie au cotrimoxazole [11]. Gesesew *et al*. en Ethiopie relevaient également le fait que la proximité aux méthodes diagnostiques et les moyens de communication favorisent la mise précoce sous TAR et donc la survie [12]. Des 45 (24,2%) enfants qui étaient décédés au moment de l´étude. Deux tiers sont décédés avant le 6^e^ mois de suivi. Ce résultat est proche de celui retrouvé par Ebissa et Njom respectivement en Ethiopie et au Cameroun (70%) [13,14] et pourrait s´expliquer par le fait que les enfants dans notre étude ont été diagnostiqués et mis sous traitement tardivement. Toutefois, on note que la survie était meilleure après le 22^e^ mois de suivi. D´autres études ont également mentionné que, la mortalité souvent élevée au début du suivi baisse par la suite ceci s´expliquant par la survenue décalée des avantages du TAR [15].

L´âge était associé significativement à la survie (p<0,0001). En effet, les enfants de moins de 2 ans avaient moins de chances de survie que les autres. Ce résultat est différent de celui de Nyunt et Ebonyi qui n´ont trouvé aucune association entre l´âge et la survie [11,16]. Dans l´étude de Nyunt *et al*. l´âge médian était de six mois, ceci montre que les enfants dans son étude étaient majoritairement plus jeunes et précocement mis sous TAR [11]. Par contre, Judd A *et al*. dans une méta-analyse réalisée dans plusieurs pays d´Europe et d´Asie du Sud Est ont retrouvé une association entre l´âge et la survie. Dans leur étude, être dans la première année de vie était prédicteur de décès précoce [17]. La scolarisation était associée significativement à la survie (p=0,004). Cela pourrait s´expliquer par le fait que les enfants scolarisés étant plus âgés comprennent mieux l´importance du suivi et du traitement dans leur prise en charge. La présence de comorbidités liées au VIH était associée significativement à la survie (p=0,005) avec un temps moyen de survie meilleur pour les enfants qui ne présentaient pas de comorbidités. Ceci est en accord avec la littérature [18].

Une association significative a été retrouvée entre le stade clinique au moment du diagnostic et la survie (p<0,0001). La probabilité de survie diminuait au fur et à mesure que le stade clinique était avancé. Ce résultat est similaire à ceux trouvés par Ebissa *et al*. en Ethiopie [13] et Njom et Loussikila au Cameroun [14]. L´anémie était un facteur associé à la survie (p=0,003). En effet, la sévérité de l´anémie diminuait la probabilité de survie. Ce résultat est semblable à celui de Vermund *et al*. au Mozambique en 2014 et Ebissa *et al*. en Ethiopie en 2015 [13,19]. Notre résultat est cependant différent de celui de Njom et Loussikila au Cameroun [14]. Cette différence pourrait s´expliquer par le fait que la majorité de leurs patients étant issus de familles de classe sociale moyenne et élevée, la proportion d´enfants avec taux d´hémoglobine <8gr/dl était faible (9%) dans leur étude [14].

Le ravitaillement en ARV était associé significativement à la survie (p<0,0001). En effet, la littérature nous rapporte que la bonne adhérence au TAR diminue la mortalité et la morbidité liée au VIH [20]. Notre résultat corrobore avec celui d´Ebissa *et al*. en Ethiopie qui retrouvait que la mauvaise adhérence au TAR était un facteur prédictif indépendant de décès [13]. Une association significative entre les maladies opportunistes et la survie a été retrouvée (p<0,0001). La probabilité de survie était meilleure pour les enfants qui n´avaient pas de maladies opportunistes. Ce résultat est comparable à celui trouvé par Traisathit *et al*. en Thaïlande dans une étude portant sur les évènements définissant le sida et décès sous antirétroviraux chez les enfants et les adolescents infectés par le VIH [21].

L´âge <2 ans [HR= 15,62 (3,1-79,7), p=0,001], l´anémie sévère [HR= 11,5 (1,1-125,3), p=0,04] et la présence des infections opportunistes [HR= 2,33 (0,98-5,5), p=0,05] étaient des facteurs indépendants significativement associés à la survie des enfants de 6 mois à 15 ans révolus infectés par le VIH à Ebolowa. Njom et Loussikila au Cameroun en 2017 a également retrouvé que l´âge ≤1 an à l´initiation du TAR était un facteur indépendant significativement associé à la diminution des chances de survie (HR= 2,1 [1,1-5,08], p=0,01), par contre, elle n´a pas retrouvé d´association significative concernant l´anémie [14]. Gebremedhin *et al*. en Ethiopie en 2013 a retrouvé comme facteurs associés diminuant significativement les chances de survie: l´âge <18 mois (HR= 4,39 [1,15-17,41], p=0,036), la diarrhée chronique (HR= 4,63 [1,50-14,31, p=0,008] et un taux d´hémoglobine <8g/dl (HR= 3,77 [29-10,98], p=0,015) [22]. Ebissa *et al*. en 2015 en Ethiopie a quant à lui retrouvé comme déterminant de la survie le taux d´hémoglobine <7g/dl (HR: 4,08 [1,33-12,56], p=0,014) [13].

## Conclusion

Au terme de cette étude, le taux de survie était de 66,7%. L´âge <2 ans, l´anémie sévère et la présence d´affections opportunistes au début du traitement antirétroviral se sont révélés être des déterminants de la survie des enfants âgés de 6 mois à 15 ans, infectés par le VIH et suivis dans la ville d´Ebolowa de 2008 à 2018. Cette étude a montré qu´en plus du traitement antirétroviral débuté précocement, un bon suivi clinique et paraclinique s´avère nécessaire pour l´amélioration de la survie des enfants infectés par le VIH.

### Etat des connaissances sur le sujet

- Le traitement antirétroviral immédiat débuté en 2011 a permis de diminuer l´incidence et la mortalité liée au VIH/SIDA et a amélioré la survie des enfants infectés;
- Très peu d´études se sont intéressées à la survie des enfants infectés par le VIH au Cameroun.

### Contribution de notre étude à la connaissance

- Cette étude montre que les taux de survie malgré la disponibilité du traitement antirétroviral restent faibles chez les enfants infectés par le VIH au Cameroun;
- L´âge <2 ans, l´anémie sévère et la présence d´affections opportunistes au début du traitement antirétroviral sont indépendamment et significativement associés à la survie des enfants infectés par le VIH au Cameroun;
- En plus du traitement antirétroviral débuté précocement, un bon suivi clinique et paraclinique s´avère nécessaire pour l´amélioration de la survie des enfants infectés par le VIH.

## Remerciements

Nos remerciements s´adressent à tous les patients et parents pour leur participation à l´étude, ainsi qu´au personnel des formations sanitaires retenues pour l´étude.
